# Metabolomics Combined with Correlation Analysis Revealed the Differences in Antioxidant Activities of Lotus Seeds with Varied Cultivars

**DOI:** 10.3390/foods13071084

**Published:** 2024-04-01

**Authors:** Xinjin Yu, Yuting Wang, Xiaoli Yan, Tuo Leng, Jianhua Xie, Qiang Yu, Yi Chen

**Affiliations:** State Key Laboratory of Food Science and Resources, Nanchang University, Nanchang 330047, China

**Keywords:** lotus seeds cultivars, UPLC-Q/TOF-MS, chemical composition, chemometrics, phenolics, antioxidant activity

## Abstract

Functional foods have potential health benefits for humans. Lotus seeds (LS) as functional foods have excellent antioxidant activities. However, the differences in chemical composition of different LS cultivars may affect their antioxidant activities. This study comprehensively analyzed the differences among five LS cultivars based on metabolomics and further revealed the effects of metabolites on antioxidant activities by correlation analysis. A total of 125 metabolites were identified in LS using UPLC-Q/TOF-MS. Then, 15 metabolites were screened as differential metabolites of different LS cultivars by chemometrics. The antioxidant activities of LS were evaluated by DPPH^•^, FRAP, and ABTS^•+^ assays. The antioxidant activities varied among different LS cultivars, with the cultivar Taikong 66 showing the highest antioxidant activities. The correlation analysis among metabolites and antioxidant activities highlighted the important contribution of phenolics and alkaloids to the antioxidant activities of LS. Particularly, 11 metabolites such as *p*-coumaric acid showed significant positive correlation with antioxidant activities. Notably, 6 differential metabolites screened in different LS cultivars showed significant effects on antioxidant activities. These results revealed the important effects of phytochemicals on the antioxidant activities of different LS cultivars. This study provided evidence for the health benefits of different LS cultivars.

## 1. Introduction

Lotus seeds (LS) are the seeds of lotus (*Nelumbo nucifera* Gaertn.), which are widely consumed because of their delicious flavor and great nutritional value [1]. LS are widely distributed and cultivated in Asia, America, and Oceania, with a history of more than 7000 years as vegetables or functional foods [2,3]. LS has been approved as “both foods as well as medicine” in China [3]. The annual production of LS in China is about 45,000 metric tonnes, contributing significant economic value [4]. With the development of the LS industry, the nutrients and functional activities of LS have been extensively investigated.

LS is rich in major food components (starch, protein, lipids, etc.) and bioactive components. Starch and protein are the main nutrients in dried LS, with the starch content exceeding 60% and a protein content of about 20% [4]. The bioactive compounds in LS mainly include flavonoids, alkaloids, phenolic acids, polysaccharides, essential oils, etc., which make LS have a wide range of biological activity [3]. LS has been reported to have effects such as antitumor [5], anti-obesity [6], anti-inflammatory [7], and hypoglycemic properties [4]. Recently, the potential health benefits of LS for humans, especially the antioxidant effects, have attracted special attention from researchers [8,9,10,11]. During domestication and breeding, LS cultivars with different characteristics were produced to improve the yield, nutritional value, taste, or functional properties. For example, the LS cultivar of Taikong 36 produced by mutagenesis in space had a higher yield and eating quality than traditional LS [12]. A previous study on 30 LS cultivars revealed significant differences in nutrient composition, such as starch, soluble sugars, and protein [13]. These studies suggested that the chemical composition of LS may largely depend on the cultivar. However, the effect of differences in the chemical composition of different LS cultivars on their antioxidant activities is not clear. Therefore, it was significant to comprehensively analyze the differences in chemical composition and antioxidant activities of different LS cultivars, and further to reveal the chemical composition affecting the differences in antioxidant activities of LS.

Metabolomics are a valuable tool for assessing the complete metabolite profiles and specific differences between species with similar characteristics [14]. This analytical method simultaneously supports studying hundreds of metabolites with high detection capabilities, allowing for the detection of small changes in compounds at low concentrations [15]. It provides an opportunity to comprehensively analyze the differences in the chemical composition of different LS cultivars. In addition, the integrated analysis of metabolomics and bioactivity can be used to elucidate the correlation among metabolites and bioactivities to identify the metabolite basis of the corresponding bioactivities [16]. Thus, metabolomics can be used to explain the differences in the antioxidant activities of different LS cultivars from the perspective of metabolites. Currently, metabolomics has been used to explore metabolite changes during LS maturation [17,18] and to characterize the metabolites in different parts of the lotus [10]. Unfortunately, there are only few studies on the metabolomics and antioxidant activities of different LS cultivars.

In summary, this study aimed to comprehensively analyze the differences among five LS cultivars based on metabolomics, and further reveal the effects of metabolites on the antioxidant activities by correlation analysis. Compared with previous studies, this study explained the differences in the functional activities of different LS cultivars from the viewpoint of phytochemicals. The results of this study provided evidence for the health benefits of LS, as well as a reference for LS cultivar breeding.

## 2. Materials and Methods

### 2.1. Chemicals Reagents

HPLC-grade acetonitrile and methanol were purchased from Merck (Darmstadt, Germany). The phenolic and organic acid standards used for UPLC-QqQ-MS analysis were all HPLC-grade and purchased from Shanghai Yuanye Bio-technology Co., Ltd. (Shanghai, China). Ethanol, sodium carbonate, cupric sulfate, potassium sulfate, sulphuric acid, sodium acetate, and ferric chloride were purchased from Xilong Science Co., Ltd. (Shanghai, China). The folin-phenol reagent was purchased from Solarbio Science and Technology Co., Ltd. (Beijing, China). 1, 1-Diphenyl-2-picrylhydrazyl (DPPH^•^) was purchased from Sinopharm (Beijing, China). 2, 2′-azino-bis (3-ethylbenzthiazoline-6-sulfonic acid (ABTS^•+^) was purchased from Beyotime Biotechnology Co., Ltd. (Shanghai, China). The Total Starch Assay Kit was purchased from Nanjing Jiancheng Bioengineering Institute Co., Ltd. (Nanjing, China). Unless specifically stated, all of the chemicals were of analytical grade.

### 2.2. Plant Materials

Five LS cultivars, including Jianxuan 17 (JX17), Taikong 36 (TK36), Taikong 66 (TK66), Baihua (BH), and Baiye (BY), were cultivated at Guangchang Bailian Institute in Jiangxi Province, China (26.79° N, 116.31° E). all of the LS cultivars were grown outdoors and cultivated under the same conditions. all of the LS samples were harvested in July 2021. The appearances of these LS cultivars are shown in Figure S1, and there were few physical differences among them. For each cultivar, six biological replicates were sampled at different locations in a field. After harvesting, the LS was shelled, peeled, and immediately transported back to the laboratory on dry ice refrigeration. After lyophilization, all of the samples were powdered in a pulverizer (JYL-CO12, Joyoung Co., Ltd., Jinan, China) and stored at −20 °C until analysis.

### 2.3. Main Chemical Composition Analysis

The moisture content of LS was determined by the direct drying method according to the Chinese national standard GB5009.3-2016 [19] The total starch content of LS was measured using the Total Starch Assay Kit following the instructions of the manufacturer. The protein content of LS was determined according to the Chinese National Standard GB5009.5-2016 [20] using an automatic Kjeldahl nitrogen tester (K9860, Hanon Scientific Instruments Co., Ltd., Jinan, China). The protein content was calculated by multiplying the nitrogen (N) content by a factor of 6.25 [21]. The fat content of LS was determined using the Soxhlet extraction method following the Chinese National Standard GB5009.6-2016 [22]. The fatty acid composition of LS was determined by referring to the method of Chen et al. [23]. The total phenolic content (TPC) of LS was determined by the Folin−Ciocalteu colorimetric method, and the results were calculated as milligrams of gallic acid equivalent per gram of dry weight of LS (mg GAE/g DW) [24].

### 2.4. Metabolomics Analysis by UPLC-Q/TOF-MS

#### 2.4.1. Extraction of Metabolites from LS

The metabolites of LS were extracted by ultrasonic-assisted solvent extraction [25]. Briefly, 7.5 mL of ethanol/water solution (7:3, *v*/*v*) was added to 0.5 g of LS powder and the mixture was vortexed for 30 s to mix fully. Subsequently, the mixture was extracted in a water bath at 60 °C for 40 min with the assistance of ultrasound at 600 W. The mixture was then centrifuged at 9168× *g* for 20 min and the supernatant was collected. The quality control (QC) sample was made by mixing equal volumes of each sample. The supernatant was filtered through a 0.22 μm polytetrafluoroethylene membrane filter before further analysis.

#### 2.4.2. The Conditions of UPLC-Q/TOF-MS Analysis

The analysis of metabolites in LS was performed on an Agilent 1290 UPLC system coupled to an Agilent 6538 Q-TOF mass spectrometer (Agilent, Santa Clara, CA, USA). An Agilent Eclipse Plus-C18 column (2.1 mm × 100 mm, 1.8 μm, Agilent Technologies, Santa Clara, CA, USA) was used for the separation of metabolites at 35 °C. The mobile phase was composed of 0.1% formic acid in water (A) and acetonitrile (B) with a flow rate of 0.3 mL/min. The injection volume was 5 μL. The gradient elution procedures were as follows: 0–5 min, 5–9% B; 5–8 min, 9–10% B; 8–9 min; 10–15% B; 9–15 min, 15–45% B; 15–23 min, 45–100% B; 23–27 min to maintain 100% B.

ESI was performed in positive ion (PI) and negative ion (NI) modes with the following ESI source operating parameters: drying gas (N_2_) flow rate, 8 L/min; gas temperature, 350 °C; nebulizer, 45 psig; sheath gas flow rate, 11 L/min; sheath gas temperature, 350 °C; Vcap, 4 kV in PI mode and 3.5 kV in NI mode; Scan range, *m*/*z* 100–1500. The reference masses were at *m*/*z* 121.0509 and 922.0098 in the PI mode and at *m*/*z* 112.9856 and 1033.9881 in the NI mode, respectively. The QC samples were added to the sample queue to assess the stability of the system and the accuracy of the experimental results.

#### 2.4.3. Data Preprocessing

The raw data files were converted to Analysis Base File format to ensure format compatibility and quick data retrieval. After that, MS-DIAL 5.1 software was used for the preprocessing of the UPLC-Q/TOF-MS data for peak alignment, peak picking, and peak annotation [26]. The exact mass tolerances for MS1 and MS2 were set at 0.01 and 0.05 Da, respectively. For peak alignment, the QC file was designated as the reference with an MS1 tolerance of 0.015 Da and a retention time tolerance of 0.05 min. The MSP format libraries used for metabolite identification were GNPS and Public EXP. For metabolite identification, manual checking and modification of the automated identification results were required to minimize the false positive rate. Finally, the data matrix derived from MS-DIAL was imported into MetaboAnalyst 5.0 for the missing value estimation, data filtering, data normalization, and chemometrics analysis. The relative content of metabolites in different LS cultivars was evaluated based on peak area normalization [27].

### 2.5. UPLC-QqQ-MS Analysis

The quantitative analysis of metabolites in LS was conducted using an Agilent 1290 UPLC system coupled with an Agilent 6430 QqQ mass spectrometer (Agilent, Santa Clara, CA, USA). An Agilent Zorbax Eclipse plus C18 column (4.6 mm× 100 mm, 1.8 μm, Agilent Technologies, USA) was used for the separation of metabolites at 35 °C. The mobile phase was composed of 0.1% formic acid in water (A) and methanol (B) with a flow rate of 0.3 mL/min. The injection volume was 3 μL. The gradient elution procedures were as follows: 0–3 min, 5–20% B; 3–8 min, 20–20% B; 8–12 min; 20–30% B; 12–13 min, 30–40% B; 13–15 min, 40–40% B; 15–17 min, 40–50% B; 17–19 min; 50–80% B; 19–22 min, 80–5% B; 22–26 min, 5–5% B. The quantification of metabolites was performed using multiple reaction monitoring (MRM) mode and negative mode ESI source. The MS/MS parameters for all analyses are shown in Table S1.

### 2.6. Antioxidant Activities Evaluation

#### 2.6.1. DPPH^•^

The free radical scavenging ability of LS extract was determined using the DPPH^•^ free radical scavenging method. Then, 40 μL of the extract (diluted five times) and 200 μL of methanol DPPH^•^ solution (0.01% *w*/*v*) were mixed and the absorbance (*A*_0_) was measured at 517 nm after being left at room temperature for 30 min in the dark. Following the appeal reaction process, the absorbance of the methanol and methanol DPPH^•^ solution was recorded as *A*_1_, and the absorbance of the extract and methanol was recorded as *A*_2_. The DPPH^•^ radical scavenging rate was calculated according to the following equation:

$${DPPH}^{•}\;radical\;scavenging\;rate=\left(1-\frac{A_{0}-A_{2}}{A_{1}}\right)\times 100\%$$

The standard curve was made using the ascorbic acid equivalent (AAE), and the calculation was expressed as µg AAE/g DW.

#### 2.6.2. Ferric Reducing Antioxidant Power (FRAP)

The FRAP reagent was prepared according to the previous method [28]. Then, 10 μL of the extract (diluted five times) and 300 μL of FRAP reagent were mixed and the absorbance was measured at 593 nm after 4 min of reaction in the dark at room temperature. The standard curve was made using FeSO_4_ solution, and the calculation was expressed as µg FeSO_4_/g DW.

#### 2.6.3. ABTS^•+^

The ABTS^•+^ radical cation scavenging activity of LS extract was assessed following the instructions of the Beyotime Institute of Biotechnology. The standard curve was made using Trolox, and the calculation was expressed as µM Trolox/g DW.

### 2.7. Statistical Analysis

The chemometrics analysis was performed on MetaboAnalyst 5.0 (https://www.metaboanalyst.ca, accessed on 1 June 2023). The PLS-DA model was evaluated by cross-validation and permutation tests (*n* = 200) at SIMCA-P 14.1. Peak areas were normalized to evaluate the relative content of metabolites, and the heatmap analysis was conducted in the OmicStudio tool (https://www.omicstudio.cn, accessed on 8 March 2024). The correlation analysis among the relative content of metabolites and antioxidant activities was also conducted in the OmicStudio tool (https://www.omicstudio.cn, accessed on 8 March 2024). The correlation was calculated by Pearson. Six biological replicates were used for all of the experiments in this study, and the results were presented as mean ± standard deviation. The significance analysis was performed using SPSS 19.0 software, and significant differences were defined as *p* < 0.05 by Tukey’s test.

## 3. Results and Discussion

### 3.1. Analysis of the Composition of Different LS Cultivars

#### 3.1.1. Main Chemical Composition Analysis

The main nutrient contents of different LS cultivars are shown in Table 1. The moisture content in fresh samples among different LS cultivars differed slightly, with the content ranging from 57.00 to 61.49 g/100 g FW, which was similar to the previous finding of Tu et al. [29]. The high moisture content provided fresh LS with the same fresh, crisp taste and active metabolism as other fruits and vegetables, which resulted in them often being eaten directly as a fruit. As an essential carbohydrate fraction, the total starch accounted for a large proportion of the dried LS, but it differed significantly among LS cultivars. The total starch content of JX17 was the highest at 72.79 g/100 g DW, while that of TK36 was the lowest at 61.34 g/100 g DW. Similarly, Sun et al. also reported that JX17 was an LS cultivar with a high starch content [13]. The protein content of LS also varied significantly depending on the cultivars in our results. TK36 had the highest protein content (23.64 g/100 g DW), while BY had the lowest (20.19 g/100 g DW). Punia Bangar et al. reported that the protein content of LS was in the range of 16–28% [4]. Zeng et al. showed that the protein in LS had a balanced amino acid composition and high protein utilization, making it an ideal source of high-quality vegetable protein [30]. In addition, the fat content of different LS cultivars was only in the range of 1.56 to 2.04 g/100 g DW, which was in line with the characteristics of high protein and low fat in LS. Phenolics are produced by the secondary metabolism of plants, which have potential antioxidant activities and may lower the risk of chronic diseases [31]. TK66 had the highest TPC (80.60 mg GAE/100 g DW) while BH had the lowest (70.75 mg GAE/100 g DW), indicating that the TPC differed depending on the LS cultivars. Different growth environments, agronomic conditions, and tissue types all resulted in differences in phenolic content, while in this study, difference in cultivar was the main reason.

#### 3.1.2. Fatty Acid Composition

As shown in Table 2, a total of 12 fatty acids were identified, of which the content of unsaturated fatty acids was approaching 80%, and polyunsaturated fatty acids reached more than 60%. C18:2 and C16:0 were the most highly abundant unsaturated and saturated fatty acids, respectively, which was consistent with the findings of Luo et al. [21]. The highest content of unsaturated and polyunsaturated fatty acids was found in TK66, which was attributed to the significantly higher content of C18:2 in TK66. It has been reported that C18:2 could be inverted into omega-3 fatty acids in vivo and contribute to the reduction of low-density lipoprotein levels in the body [4]. These results suggested that the fatty acid composition varied among the LS cultivars. Fatty acids are vital nutrients and metabolites in organisms. Unsaturated fatty acids have been proven to be beneficial to humans, while an excessive intake of saturated fatty acids can increase the risk of some chronic diseases [32]. Therefore, the appropriate ratio of unsaturated to saturated fatty acids in food is important for human health. TK66 had the highest percentage of unsaturated fatty acids among all of the tested cultivars, which might be more beneficial to human health.

### 3.2. Metabolomics Analysis of Different LS Cultivars Based on UPLC-Q/TOF-MS

#### 3.2.1. Metabolites Analysis of LS

**Figure 1:**
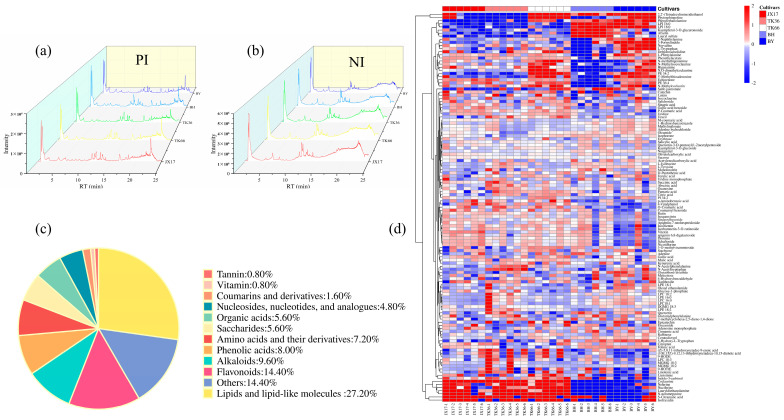
Total ion chromatograms of different LS cultivars in positive ions (**a**) and negative ions (**b**). Classification of metabolites identified in LS (**c**). Heatmap analysis of the relative content of metabolites in different LS cultivars (**d**).

The metabolites in LS were detected by UPLC-Q/TOF-MS in both PI and NI modes. The total ion chromatograms (TIC) of different LS cultivars are presented in Figure 1. The metabolites were presumptively annotated using databases and the relevant literature based on information such as retention times, parent ions, and secondary fragment ions. Here, 71 and 81 metabolites were identified in PI and NI modes, respectively. The ion mass spectra of some representative metabolites were selected and are presented in Figure S2. A total of 125 metabolites were identified after combining the PI and NI models, including 10 phenolic acids; 7 organic acids; 18 flavonoids; 9 amino acids and their derivatives; 12 alkaloids, 34 lipids and lipid-like molecules, 7 saccharides, 6 nucleosides, nucleotides, and analogues; 2 coumarins and derivatives, 1 vitamin, 1 tannin, and 18 other metabolites (Figure 1c and Table S2). The relative content of these metabolites in different LS cultivars is shown in a heat map (Figure 1d). It was found that the phenolic acids and organic acids were highly abundant in TK66, the flavonoids were abundant in JX17, and the alkaloids were abundant both in TK66, TK36, and JX17. In addition, it was worth noting that BY was relatively lower in the content of organic acids, flavonoids, and alkaloids, while it was rich in amino acids and lipids. Previous LC−MS studies on LS mainly focused on specific metabolites, such as phenolics [10], oligomeric proanthocyanidins, alkaloids, and flavonoids [18]. Compared with previous studies, this study described a comprehensive and comparative LC−MS metabolic profile of five LS cultivars for the first time, incorporating chemometrics to investigate the variation in secondary metabolites among cultivars, thus providing better chemical evidence for the nutritional and health benefits of LS.

#### 3.2.2. Principal Component Analysis (PCA)

PCA is commonly employed to synthetically analyze the clustering trends in multidimensional data [27]. PCA was employed to initially evaluate the clustering and the differences in metabolite composition among different LS cultivars (Figure S3). The QC samples were tightly clustered in the PCA score plot, demonstrating that the LC−MS analysis had excellent reproducibility and stability. In PI mode, the first two principal components (PCs) explained 27.8% of the variance among the test samples (17.2% in PC1 and 10.6% in PC2). TK36 was separated from the other LS cultivars in the PCA score plot, indicating that TK36 was greatly different in metabolite composition from the other LS cultivars in PI mode. While in the NI mode, the first two principal components (PCs) explained 52.5% of the variance among the test samples (37.8% in PC1 and 14.7% in PC2). BH was separated from the other LS cultivars in the PCA score plot, indicating that BH was greatly different in metabolite composition from the other LS cultivars in NI mode. The results suggested that the metabolites of different LS cultivars were similar and PCA was able to distinguish them to some extent.

#### 3.2.3. Partial Least Squares-Discriminant Analysis (PLS-DA)

**Figure 2:**
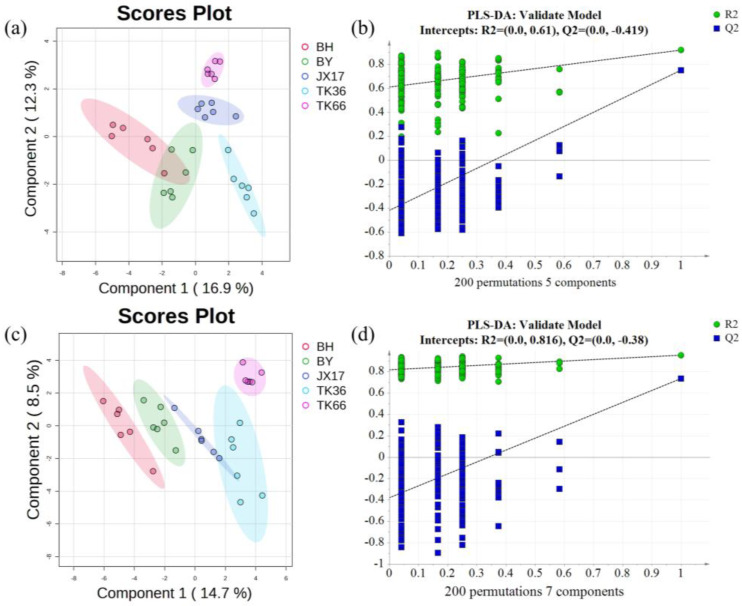
The PLS-DA score plots for different LS cultivars: (**a**) positive ion mode and (**c**) negative ion mode. The permutation test results of the PLS-DA model: (**b**) positive ion mode and (**d**) negative ion mode.

It is important to build an accurate discriminatory model for screening and identifying key metabolites in large metabolomics datasets. To further reflect the differences in metabolites among LS cultivars and to screen for the differential metabolites, a PLS-DA analysis was conducted. PLS-DA is a supervised data analysis approach that can be used to distinguish among different classes [33]. Compared with the results of PCA, the different LS cultivars showed good separation in the PLS-DA score plot in both PI and NI modes (Figure 2). It was notable that BY and BH were still partially overlapping in the score plot in the PI mode, probably due to the strong similarity in metabolite composition between the two as traditional local cultivars in Guangchang. The parameters of the PLS-DA model developed based on the metabolite ion characteristics of the PI and NI modes are shown in Table S3. The R^2^ and Q^2^ of the PLS-DA model were >0.5 for both PI and NI modes, indicating that the models were reliable and with an excellent predictive power [27]. In addition, the cross-validation and permutation tests (200 times) were conducted to evaluate whether the models were over-fitted. The P_CV-ANOVE_ was <0.05, and the R^2^ and Q^2^ of the random model decreased as the permutation retention decreased (Figure 2), suggesting that the original models were not over-fitted and were robust [27]. It was demonstrated that the PLS-DA models based on the metabolite ion characteristics possessed a good predictive performance and could be reliably used to screen for differential metabolites in different LS cultivars.

**Figure 3:**
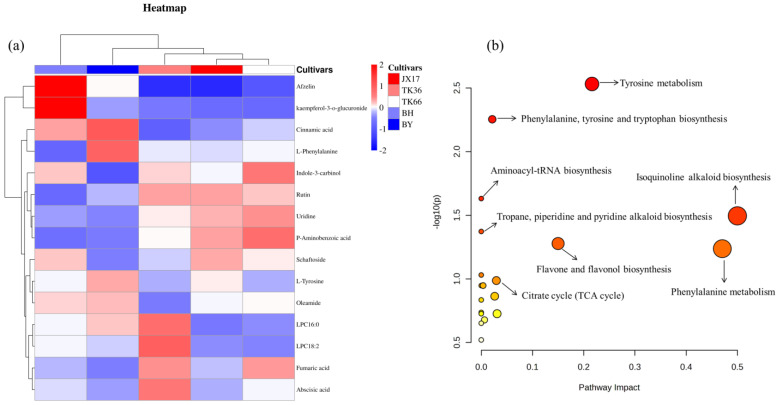
Heatmap analysis based on the relative content of 15 differential metabolites (**a**). Pathway enrichment analysis of the 15 differential metabolites (**b**).

The variable importance of projections (VIP) in the PLS-DA model is often employed as an important index for mining biologically significant differential metabolites. In this study, variables with VIP > 1.0 and *p* < 0.05 (ANOVA) were considered differential metabolites in different LS cultivars [27]. As a result, there were 15 differential metabolites screened in different LS cultivars, of which 10 were screened in PI mode and 5 in NI mode. These differential metabolites included four flavonoids, four lipids, two organic acids, two amino acids, one benzoic acid derivative, one pyrimidine, and one indole derivative (Table S4). The relative contents of these 15 differential metabolites in different LS cultivars are shown in Figure 3a.

Four flavonoids were screened as differential metabolites in this study, including rutin, schaftoside, afzelin, and kaempferol-3-*O*-glucuronide. Flavonoids are beneficial to humans because they exhibit a wide range of biological activity. Rutin is found in many plants and possesses various biological activities such as antioxidant, neuroprotective, anti-inflammatory, nephroprotective, and hepatoprotective effects [34]. The previous study showed that the rutin content also differed significantly in different buckwheat cultivars, which was related to the expression level of the flavonoid biosynthesis gene Flavonol synthase 1 [35]. Schaftoside is a *C*-glycosyl flavonoid, which has been shown to have anti-inflammatory [36] and nonalcoholic fatty liver prevention effects [37]. Afzelin is a flavonol originally found in *Nymphaea odorata*, which has been reported to exhibit antioxidant, anti-inflammatory, anti-cancer, and antibacterial properties [38]. There were fewer studies on the biological activity of kaempferol-3-*O*-glucuronide, which has been reported to have an antiviral activity [39].

There were four lipids screened as differential metabolites in different LS cultivars; they were abscisic acid, oleamide, Lysophosphatidylcholine (LPC) 16:0, and LPC 18:2. Lipids are an essential part of the human diet and play a key role in the acceptability and flavor of foods [27]. Notably, LPC 16:0 and LPC 18:2 have also been reported as differential metabolites in different wheat cultivars [40].

There were two organic acids screened as differential metabolites for different LS cultivars, which were cinnamic acid and fumaric acid. In addition to their safety functions as preservatives, the high level of organic acids in food is also beneficial to the organoleptic properties of foods. Cinnamic acid is produced through phenylalanine deamination in plants, which has many physiological activities such as anti-proliferative, anti-inflammatory, antioxidant, and anti-tumor effects [41]. Fumaric acid is an intermediate in the citric acid cycle, which is widely used in food, beverage, pharmaceutical, and other industrial applications [42]. The results of Wang et al. showed that fumaric acid was significantly varied during LS development and was an important contributor to the developmental stage characteristics of LS [17].

Amino acids possess many nutritional and physiological functions in the human body, and they are key to the taste of foods [27]. L-phenylalanine and L-tyrosine were screened as differential metabolites in different LS cultivars in this study. The content of these two amino acids in BY was significantly higher than those of the other cultivars, highlighting the nutritional significance of BY.

The other three differential metabolites were *p*-aminobenzoic acid, uridine, and indole-3-carbinol, all of which exhibited a relatively high content in TK66. *P*-aminobenzoic acid is an essential substrate for the synthesis of many types of biological scaffolds, and it also has obvious pharmacological significance [43]. Uridine is widely used in foods and pharmaceutical industries, where it has been reported to have cardioprotective effects and to prevent hypoxic lung injury [44]. Indole-3-carbinol is a natural glucosinolate that is famous for its cancer-preventive activity. In addition, it has also been reported to have antioxidant, antibacterial, antiviral, and anti-inflammatory effects [45].

#### 3.2.4. Metabolic Pathway Analysis

Metabolic pathway enrichment analysis of the 15 differential metabolites was conducted using the KEGG database and the results are shown in a bubble chart (Figure 3b) [46]. The bubbles represented the metabolic pathways, while the horizontal coordinates and size of the bubbles represented influencing factors of the metabolic pathways. The *p*-value of the enrichment analysis was represented by the vertical coordinate and color of the bubbles. Tyrosine metabolism, phenylalanine, tyrosine and tryptophan biosynthesis, aminoacyl-tRNA biosynthesis, isoquinoline alkaloid biosynthesis, tropane, piperidine and pyridine alkaloid biosynthesis, and flavone and flavonol biosynthesis were mainly enriched pathways. These metabolic pathways were mainly associated with amino acid metabolism as well as the biosynthesis of amino acids, alkaloids, and flavonoids. These results showed that cultivars were strongly influencing the biosynthesis of secondary metabolites in LS. The differential metabolic pathways among LS cultivars need to be further identified in combination with other omics in the future.

### 3.3. UPLC-QqQ-MS Analysis

A total of three organic acids, four phenolic acids, and six flavonoids in LS were quantified by UPLC-QqQ-MS, four of which were differential metabolites of different LS cultivars screened by chemometrics analysis. The standard curves, correlation coefficients, and linear ranges for the 13 compounds are shown in Table S5. All standards showed good linearity in terms of concentration and peak area. The results of the quantitative analysis of the 13 compounds in different LS cultivars are shown in Table 3. Malic acid showed the highest content of all the compounds quantified (323.47–418.14 µg/g DW). Similar to our results, malic acid was previously reported to be the most abundant organic acid in LS [17]. In this study, rutin, isoorientin, and schaftoside were the main flavonoids in LS. A previous study showed that the rutin and schaftoside content of JX17 at maturity was about 3.42 and 5.09 µg/g DW, respectively [18]. In contrast, higher contents of rutin and schaftoside were measured in this study, which might be due to the different origins and extraction methods. The catechin content of LS ranged from 1.99 to 2.89 µg/g DW. Yu et al. reported that catechin was gradually reduced during the development of LS, which is one of the major proanthocyanidins in LS [18]. Furthermore, BY showed lower content in most phenolics than the other cultivars, this was consistent with the result of its lower TPC. Fumaric acid, rutin, schaftoside, and cinnamic acid were the differential metabolites of different LS cultivars screened in chemometrics. The results of the UPLC-QqQ-MS analyses showed that their contents differed significantly among LS cultivars. In addition, the differences in their relative contents in different LS cultivars were consistent with the results of metabolomics (Figure 3a), which verified the accuracy of the metabolomics analysis results. The differences in the phenolic content of the different LS cultivars might signal differences in their functional activities.

### 3.4. Antioxidant Activities of Different LS Cultivars

**Figure 4:**
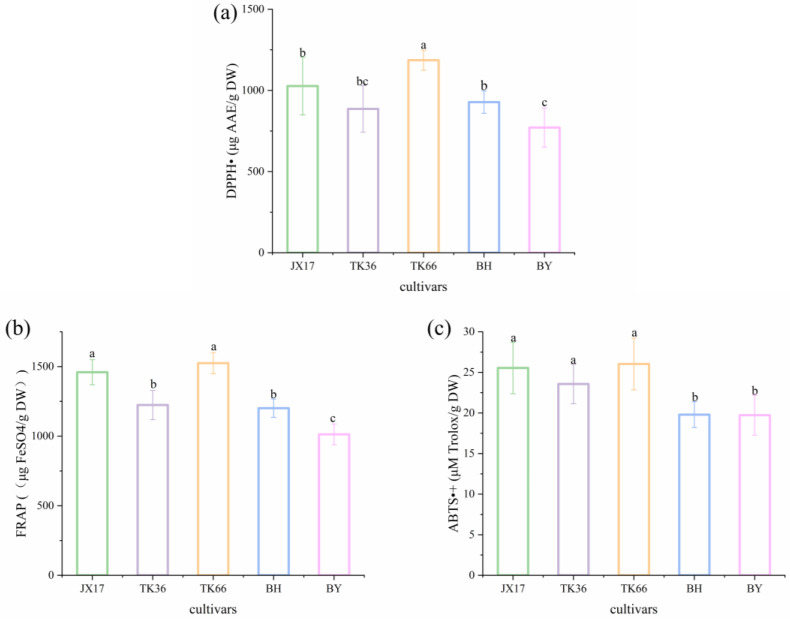
The antioxidant activities of different LS cultivars: (**a**) DPPH^•^, (**b**) FRAP, (**c**) ABTS^•+^. Values with different letters show a significant difference (*p* < 0.05).

As a popular functional food, LS is rich in bioactive compounds such as phenolics, flavonoids, and alkaloids, which have a potential antioxidant capacity [8]. Therefore, the antioxidant capacity of different LS cultivars was assessed by employing DPPH^•^, FRAP, and ABTS^•+^ (Figure 4). Among the different LS cultivars, TK66 showed the strongest DPPH^•^ free radical scavenging activity (1186.09 μg AAE/g DW), followed by JX17 (1026.83 μg AAE/g DW), while BY was the lowest (771.09 μg AAE g DW). The FRAP-reducing capacity of different LS cultivars ranged from 1013.19 to 1524.74 μg FeSO_4_/g DW, and the antioxidant capacity of TK66 and JX17 was significantly higher than that of the other cultivars. ABTS^•+^ is also an effective method for determining the antioxidant activities of LS. The ABTS^•+^ radical scavenging activity of different LS cultivars ranged from 19.73 to 26.04 μM Trolox/g DW. The antioxidant capacity of TK66, JX17, and TK36 was significantly higher than that of the other cultivars. Although the principles of the three antioxidant capacity test methods were different, TK66 showed a higher antioxidant capacity regardless of which test method was used, while BY showed the lowest antioxidant capacity. The free radical scavenging activities of LS may be realized by providing electron or hydrogen donors through LS extract. The antioxidant mechanism may be due to the supply of hydrogen by the antioxidant, which binds to the free radicals and forms stabilized radicals to terminate the free radical chain reaction or combine with free radical ions [47]. In addition, the reducing activity of antioxidants is usually achieved by giving away hydrogen atoms or destroying free radical chains. Phenolics and alkaloids have been reported to be effective at scavenging free radicals and reducing metal ions, which may be the main contributors to the antioxidant activities of LS [48]. The lower antioxidant capacity of LS was measured in this study compared with the previous study [10], which was probably caused by various factors such as the origin of the samples and the extraction method. In conclusion, LS possessed strong antioxidant activities, but there were significant differences among cultivars. This was probably a result of the different contents of antioxidants in the extracts of different LS cultivars [32]. Therefore, an in-depth investigation into the correlation among LS metabolites and antioxidant activities could help explain the differences in antioxidant activities among different LS cultivars.

### 3.5. Correlation Analysis of Chemical Composition and Antioxidant Activities

**Figure 5:**
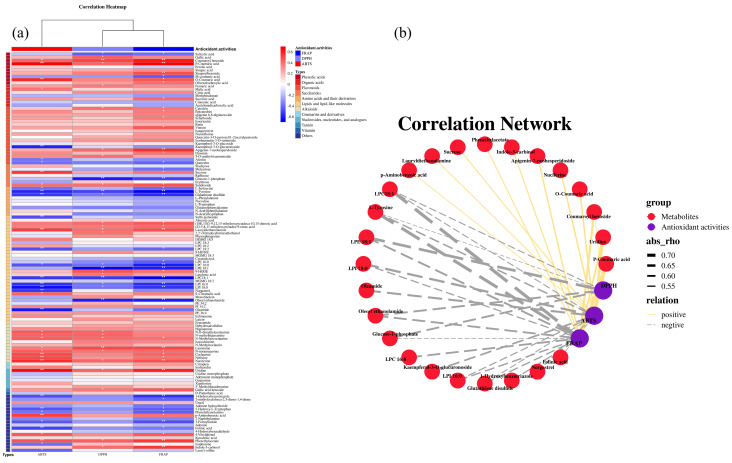
The correlation heatmap (**a**) and correlation network (**b**) among antioxidant activities and the relative content of metabolites. * indicated *p* < 0.05; ** indicated *p* < 0.01.

To investigate the effect of chemical composition in LS on their antioxidant activities, a correlation heatmap of metabolites’ relative content and antioxidant activities in different LS cultivars was conducted (Figure 5a) [49]. It revealed that the antioxidant activities were positively correlated with metabolites such as phenolic acids, flavonoids, and alkaloids, while negatively correlated with amino acids and some lipids. Phenolics and alkaloids possessed strong antioxidant activities, which were effective in scavenging free radicals and reducing metal ions [48]. Thus, the relatively high content of phenolics and alkaloids detected in TK66, TK36, and JX17 might contribute to their high antioxidant activities.

To clarify the metabolites that were highly correlated with the antioxidant activities in LS, metabolites with |r| > 0.5 and *p* < 0.05 were further screened, and a correlation network among metabolites and antioxidant activities was established (Figure 5b). The results showed that 25 metabolites were significantly and closely correlated with antioxidant activities. Among them, 11 metabolites showed significant positive correlation with antioxidant activities including *p*-coumaric acid, uridine, coumaroyl hexoside, *o*-coumaric acid, nuciferine, apigenin-7-neohesperidoside, indole-3-carbinol, phenethylacetate, sucrose, lauryldiethanolamine, and *p*-aminobenzoic acid. The antioxidant activities of most of these metabolites have been reported. Both *p*-coumaric acid and *o*-coumaric acid were reported to be the major contributors to the antioxidant activities of free phenols in gluten-free flakes [50]. Similarly, the *p*-coumaric acid was highly correlated with the antioxidant activity in this study. The results of the UPLC-QqQ-MS analysis showed that TK66, TK36, and JX17 had a significantly higher content of *p*-coumaric acid than BY and BH, which might be the reason for their higher antioxidant activities. Apigenin-7-neohesperidoside was reported to have strong antioxidant activities and to be anti-inflammatory [51]. Nuciferine is an important pharmacological component of Lotus and has multiple biological activities. Nuciferine was reported to protect against obesity-induced nephrotoxicity through its hypolipidemic, anti-inflammatory, and antioxidant effects [52]. In addition, *p*-aminobenzoic acid, uridine, and indole-3-carbinol were reported to possess significant antioxidant activities [45,53,54]. It was found that the relative content of these three metabolites was significantly higher in TK66 than in the other cultivars (Figure 3a), which might contribute to the higher antioxidant activities of TK66. While 14 metabolites, such as LPC 18:1, tyrosine, LPE 18:1, and LPI 18:0, showed a significant negative correlation with antioxidant activities. Among the 25 metabolites highly correlated with antioxidant activities of LS, tyrosine, *p*-aminobenzoic acid, uridine, indole-3-carbinol, oleamide, and LPC 16:0 were also screened as differential metabolites in different LS cultivars by chemometrics. It indicated that differences in the secondary metabolites of different LS cultivars had a significant impact on their antioxidant activities. In conclusion, the difference in antioxidant activities of different LS cultivars was strongly correlated with differences in the relative content of secondary metabolites. This study explained the differences in the antioxidant activities of different LS cultivars from the viewpoint of chemical composition. LS is often consumed as a functional food, so characterizing the antioxidant activities of different LS cultivars would be helpful in the development of related products. The LS cultivars can be selectively grown in actual agricultural production based on their chemical composition and functional activities. In addition, a comprehensive analysis of existing LS cultivars can provide a reference for the cultivation of new LS cultivars.

## 4. Conclusions

This study comprehensively analyzed the differences in phytochemicals of five LS cultivars based on metabolomics and further revealed the effects of metabolites on the antioxidant activities of LS by correlation analysis. TK66 showed a higher unsaturated fatty acid percentage, TPC, and antioxidant activities, which may be more beneficial to human health. A total of 125 metabolites were identified in LS based on UPLC-Q/TOF-MS, and 15 metabolites were screened as differential metabolites of LS cultivars by chemometrics. The UPLC-QqQ-MS analysis showed that 10 phenolics and 3 organic acids differed among the LS cultivars, which validated the results of the chemometrics. The correlation analysis of antioxidant activities with metabolites showed that metabolites such as phenolics and alkaloids contributed to the antioxidant activities of LS. Particularly, 11 metabolites such as *p*-coumaric acid showed significant positive correlation with the antioxidant activities. In addition, six differential metabolites screened in different LS cultivars showed significant effects on the antioxidant activities. This study provided evidence for the health benefits of different LS cultivars. In the future, more bioactivities of LS should be further explored through in vivo experiments to provide a basis for the development of LS products.

## Figures and Tables

**Table 1:** The main chemical composition analysis of different LS cultivars.

Components	JX17	TK66	TK36	BH	BY
Moisture g/100 g FW	58.41 ± 1.41 ^bc^	61.49 ± 1.65 ^a^	60.64 ± 1.22 ^a^	59.71 ± 1.86 ^ab^	57.00 ± 1.98 ^c^
Total starch g/100 g DW	72.79 ± 3.77 ^a^	72.16 ± 1.63 ^a^	61.34 ± 3.98 ^b^	62.89 ± 3.88 ^b^	64.08 ± 4.44 ^b^
Protein g/100 g DW	21.35 ± 0.94 ^b^	21.71 ± 0.72 ^b^	23.64 ± 0.66 ^a^	22.76 ± 1.03 ^a^	20.19 ± 0.45 ^c^
Fat g/100 g DW	1.56 ± 0.06 ^c^	1.76 ± 0.09 ^b^	2.04 ± 0.11 ^a^	1.74 ± 0.06 ^b^	1.79 ± 0.10 ^b^
TPC mg GAE/100 g DW	73.19 ± 6.51 ^ab^	80.60 ± 4.9 ^a^	73.58 ± 5.8 ^ab^	70.75 ± 5.59 ^b^	71.24 ± 5.66 ^b^

Moisture content was calculated by fresh weight (FW), and the rest of indicators were calculated by dry weight (DW). Values with different letters show a significant difference (*p* < 0.05).

**Table 2:** Fatty acid composition analysis of different LS cultivars (%).

Fatty Acid	JX17	TK66	TK36	BH	BY
C11:0	0.08 ± 0.00 ^a^	0.06 ± 0.01 ^a^	ND	ND	0.08 ± 0.02 ^a^
C14:0	0.30 ± 0.01 ^a^	0.29 ± 0.01 ^a^	0.30 ± 0.01 ^a^	0.29 ± 0.02 ^a^	0.30 ± 0.01 ^a^
C16:0	15.66 ± 0.28 ^b^	15.42 ± 0.42 ^b^	16.74 ± 1.29 ^a^	15.98 ± 0.49 ^ab^	16.57 ± 0.69 ^a^
C16:1	0.15 ± 0.00 ^ab^	0.15 ± 0.01 ^a^	0.15 ± 0.01^b^	0.16 ± 0.01 ^a^	0.15 ± 0.01 ^b^
C18:0	2.18 ± 0.11 ^b^	2.13 ± 0.13 ^bc^	2.36 ± 0.10 ^a^	2.00 ± 0.09 ^c^	2.43 ± 0.11 ^a^
C18:1	11.33 ± 0.79 ^bc^	9.90 ± 0.26 ^c^	11.01 ± 1.27 ^bc^	13.00 ± 2.60 ^b^	16.50 ± 3.93 ^a^
C18:2	53.79 ± 1.10 ^ab^	55.03 ± 0.56 ^a^	52.99 ± 1.19 ^b^	52.89 ± 1.78 ^b^	48.52 ± 2.41 ^c^
C20:0	2.10 ± 0.04 ^bc^	2.45 ± 0.11 ^a^	2.23 ± 0.28 ^b^	1.94 ± 0.19 ^c^	2.62 ± 0.20 ^a^
C18:3	5.50 ± 0.35 ^ab^	5.33 ± 0.25 ^b^	5.50 ± 0.33 ^ab^	5.80 ± 0.37 ^a^	3.60 ± 0.24 ^c^
C20:3	6.93 ± 0.28 ^a^	7.25 ± 0.38 ^a^	6.86 ± 0.70 ^a^	6.11 ± 0.66 ^b^	7.38 ± 0.63 ^a^
C20:4	0.20 ± 0.02 ^ab^	0.20 ± 0.01 ^ab^	0.22 ± 0.02 ^a^	0.18 ± 0.03 ^b^	0.20 ± 0.01 ^ab^
C22:2	1.83 ± 0.13 ^a^	1.80 ± 0.08 ^a^	1.68 ± 0.20 ^a^	1.64 ± 0.15 ^a^	1.65 ± 0.17 ^a^
SFA	20.30 ± 0.31 ^b^	20.34 ± 0.43 ^b^	21.64 ± 0.96 ^a^	20.22 ± 0.58 ^b^	22.00 ± 0.77 ^a^
UFA	79.70 ± 0.31 ^a^	79.66 ± 0.43 ^a^	78.36 ± 0.96 ^b^	79.78 ± 0.58 ^a^	78.00 ± 0.77^b^
PUFA	68.26 ± 1.09 ^ab^	69.61 ± 0.41 ^a^	67.25 ± 0.69 ^ab^	66.62 ± 2.40 ^b^	61.35 ± 3.23 ^c^
UFA/SFA	3.93 ± 0.07 ^a^	3.92 ± 0.11 ^a^	3.63 ± 0.20^b^	3.95 ± 0.14 ^a^	3.55 ± 0.17 ^b^

C11:0, undecanoic; C14:0, myristic acid; C16:0, palmitic acid; C16:1, palmitoleic acid; C18:0, stearic acid; C18:1, oleic acid; C18:2, linoleic acid; C20:0, arachidic acid; C18:3, linolenic acid; C20:3, eicosatrienoic acid; C20:4, eicosatetraenoic acid; C22:2, docosadienoic acid; SFA, saturated fatty acids; UFA, unsaturated fatty acids; PUFA, polyunsaturated fatty acids. Values with different letters show a significant difference (*p* < 0.05). ND: not detected.

**Table 3:** The major organic acid, phenolic acid, and flavonoid contents in different LS cultivars (µg/g DW).

Compounds	TK36	TK66	JX17	BY	BH
Malic acid	402.37 ± 25.81 ^b^	418.14 ± 41.29 ^a^	357.25 ± 39.63 ^bc^	359.76 ± 40.18 ^bc^	323.47 ± 7.19 ^c^
Fumaric acid	16.76 ± 0.79 ^a^	15.26 ± 0.66 ^ab^	14.25 ± 0.43 ^b^	15.17 ± 1.24 ^b^	14.77 ± 1.16 ^b^
Gallic acid	0.28 ± 0.03 ^b^	0.27 ± 0.02 ^ba^	0.33 ± 0.03 ^b^	0.29 ± 0.03 ^b^	0.43 ± 0.09 ^a^
Catechin	1.99 ± 0.21 ^b^	2.89 ± 0.39 ^a^	2.07 ± 0.29 ^b^	2.53 ± 0.20 ^ab^	2.40 ± 0.31 ^ab^
Epicatechin	0.26 ± 0.03 ^ab^	0.29 ± 0.04 ^a^	0.21 ± 0.03 ^c^	0.29 ± 0.03 ^ab^	0.22 ± 0.03 ^bc^
Schaftoside	7.13 ± 1.95 ^b^	6.80 ± 1.05 ^bc^	11.33 ± 1.61 ^a^	4.09 ± 0.66 ^c^	7.65 ± 1.43 ^b^
Isoorientin	3.03 ± 0.63 ^bc^	4.44 ± 1.13 ^b^	8.24 ± 1.21 ^a^	2.02 ± 0.66 ^c^	4.97 ± 1.01 ^b^
*p*-Coumaric acid	0.88 ± 0.16 ^bc^	1.36 ± 0.15 ^a^	0.91 ± 0.15 ^b^	0.60 ± 0.10 ^d^	0.64 ± 0.10 ^cd^
Sinapic acid	0.28 ± 0.02 ^ab^	0.26 ± 0.07 ^b^	0.39 ± 0.08 ^a^	0.21 ± 0.06 ^b^	0.27 ± 0.03 ^b^
Ferulic acid	0.45 ± 0.07 ^b^	0.45 ± 0.08 ^b^	0.61 ± 0.09 ^ab^	0.75 ± 0.12 ^a^	0.44 ± 0.09 ^b^
Rutin	7.80 ± 1.57 ^b^	10.61 ± 2.01 ^a^	10.04 ± 0.82 ^a^	7.50 ± 1.03 ^b^	7.86 ± 0.83 ^b^
Isoquercitrin	1.86 ± 0.44 ^ab^	1.78 ± 0.30 ^bc^	2.35 ± 0.33 ^ab^	1.53 ± 0.25 ^c^	2.32 ± 0.12 ^a^
Cinnamic acid	0.42 ± 0.07 ^bc^	0.32 ± 0.09 ^c^	0.57 ± 0.58 ^b^	0.58 ± 0.10 ^b^	0.91 ± 0.18 ^a^

Values with different letters show a significant difference (*p* < 0.05).

## Data Availability

The original contributions presented in the study are included in the article and Supplementary Material, further inquiries can be directed to the corresponding author.

## Supplementary Materials

The following supporting information can be downloaded at: https://www.mdpi.com/article/10.3390/foods13071084/s1. Table S1: The UPLC-QqQ-MS acquisition parameters for 13 standards. Table S2: The information of the compounds identified in LS. Table S3. The parameters of PLS-DA models. Table S4: The information of differential metabolites screened by PLS-DA. Table S5: The linear equation, Regression coefficient, and Linear range of 13 standards. Figure S1: Appearance of different LS cultivars (left: with shell; right: without shell). Figure S2: Selected-MS product ion spectra of Schaftoside (a), Rutin (b), Raffinose (c), Malic acid (d), Citric acid (e), and L-Tryptophan (f). Figure S3: The PCA score plots for different LS cultivars in positive ion mode (a) and negative ion mode (b).
