# Supplementary material for: Metabolomics Combined with Correlation Analysis Revealed the Differences in Antioxidant Activities of Lotus Seeds with Varied Cultivars
Source: Foods. 2024 Apr 1;13(7):1084. doi: 10.3390/foods13071084 (PMC11011491; doi:10.3390/foods13071084)
Supplement: Supplementary file 1 [file foods-13-01084-s001.zip › foods-2936228-supplementary.pdf]

**Table S1.** The UPLC-QqQ-MS acquisition parameters for 13 standards.

**Table S2.** The information of the compounds identified in LS.

**Table S3.** The parameters of PLS-DA models.

**Table S4.** The information of differential metabolites screened by PLS-DA.

**Table S5.** The linear equation, Regression coefficient, and Linear range of 13 standards.

**Figure S1.** Appearance of different LS cultivars (left: with shell; right: without shell).

**Figure S2.** Selected-MS product ion spectra of Schaftoside (a), Rutin (b), Raffinose (c), Malic acid (d), Citric acid (e), and L-Tryptophan (f).

**Figure S3.** The PCA score plots for different LS cultivars in positive ion mode (a) and negative ion mode (b).

**Table S1.** The UPLC-QqQ-MS acquisition parameters for 13 standards.

| Compounds | Formula | RT (min) | Precursor ion (m/z) | Product ion (m/z) | Fragmentor voltage (V) | Collision energy (V) |
|---|---|---|---|---|---|---|
| Malic acid | $C_4H_6O_5$ | 4.40 | 133 | 115 | 55 | 10 |
| Fumaric acid | $C_4H_4O_4$ | 6.55 | 115 | 71 | 55 | 5 |
| Gallic acid | $C_7H_6O_5$ | 7.56 | 169.1 | 125.2 | 100 | 12 |
| Catechin | $C_{15}H_{14}O_6$ | 14.84 | 289 | 125 | 125 | 14 |
| Epicatechin | $C_{15}H_{14}O_6$ | 17.45 | 289 | 109 | 135 | 22 |
| Schaftoside | $C_{26}H_{28}O_{14}$ | 20.03 | 563 | 353 | 220 | 35 |
| Isoorientin | $C_{21}H_{20}O_{11}$ | 20.30 | 447 | 327 | 140 | 20 |
| *p*-Coumaric acid | $C_9H_8O_3$ | 20.65 | 163 | 119 | 60 | 15 |
| Sinapic acid | $C_{11}H_{12}O_5$ | 20.90 | 223 | 93 | 60 | 30 |
| Ferulic acid | $C_{10}H_{10}O_4$ | 20.98 | 193.12 | 134.2 | 135 | 8 |
| Rutin | $C_{27}H_{30}O_{16}$ | 21.58 | 609 | 300 | 170 | 32 |
| Isoquercitrin | $C_{21}H_{20}O_{12}$ | 21.64 | 463 | 301 | 175 | 25 |
| Cinnamic acid | $C_9H_8O_2$ | 23.03 | 147 | 103 | 65 | 5 |

**Table S2.** The information of the compounds identified in LS.

| NO | RT | Measured (m/z) | Calulated (m/z) | (Error) /ppm | Adducts | Major fragment ions (m/z) | Formula | Identification | Compound class |
|---|---|---|---|---|---|---|---|---|---|
| 1 | 0.88 | 665.2130 | 665.2140 | 1.56 | $[M-H]^-$ | 383.1215、179.0562、485.1522、443.1415、101.0252 | $C_{24}H_{42}O_{21}$ | Stachyose | Saccharides |
| 2 | 0.88 | 543.1315 | 543.1322 | 1.23 | $[M+k]^+$ | 381.0761 | $C_{18}H_{32}O_{16}$ | Melezitose | Saccharides |
| 3 | 0.92 | 341.1103 | 341.1092 | 3.22 | $[M-H]^-$ | 89.0245、59.0143、179.0559、119.0349、71.0140、101.0246 | $C_{12}H_{22}O_{11}$ | Sucrose | Saccharides |
| 4 | 0.92 | 503.1641 | 503.1612 | 5.76 | $[M-H]^-$ | 179.0569、89.0253、221.0679、161.0463、59.0152、101.0262、116.0252 | $C_{18}H_{32}O_{16}$ | Raffinose | Saccharides |
| 5 | 1.00 | 243.0631 | 243.0617 | 5.76 | $[M-H]^-$ | 110.0241、82.0300、140.0334、152.0350、200.0530 | $C_9H_{12}N_2O_6$ | Uridine | Nucleosides, nucleotides, and analogues |
| 6 | 1.00 | 132.1023 | 132.1019 | 3.03 | $[M+H]^+$ | 86.0863、69.0618、57.515 | $C_6H_{13}NO_2$ | L-Isoleucine | Amino acids and their derivatives |
| 7 | 1.00 | 180.0663 | 180.0660 | 1.67 | $[M-H]^-$ | 119.0501、163.0407、93.0339、72.0091 | $C_9H_{11}NO_3$ | L-Tyrosine | Amino acids and their derivatives |
| 8 | 1.00 | 136.0497 | 136.0505 | 6.10 | $[M+H]^+$ | 91.0442、119.0340、53.0303 | $C_6H_5N_3O$ | 1-Hydroxybenz otriazole | Others |
| 9 | 1.03 | 145.0310 | 145.0300 | 6.69 | $[M+Na]^+$ | 84.9596 | $C_7H_6O_2$ | 2-methylcycloh exa-2,5-diene-1, 4-dione | Others |
| 10 | 1.07 | 115.0031 | 115.0023 | 6.96 | $[M-H]^-$ | 71.012 | $C_4H_4O_4$ | Fumaric acid | Organic acids |
| 11 | 1.07 | 133.0141 | 133.0137 | 3.08 | $[M-H]^-$ | 115.0036、71.0137、89.0245、72.9940 | $C_4H_6O_5$ | Malic acid | Organic acids |
| 12 | 1.11 | 259.0237 | 259.0220 | 6.60 | $[M-H]^-$ | 78.9582、96.9698、138.9783 | $C_6H_{13}O_9P$ | Glucose 1-phosphate | Saccharides |

| | | | | | | | | | |
|---|---|---|---|---|---|---|---|---|---|
| 13 | 1.15 | 323.0306 | 323.0290 | 4.83 | $[M-H]^-$ | 78.9591、96.9700、111.0211、138.9806、150.9797、211.0008 | $C_9H_{13}N_2O_9P$ | Uridine Monophosphate | Nucleosides, nucleotides, and analogues |
| 14 | 1.15 | 346.0590 | 346.0558 | 9.28 | $[M-H]^-$ | 78.9590、96.9697、134.0459 | $C_{10}H_{14}N_5O_7P$ | Adenosine monophosphate | Nucleosides, nucleotides, and analogues |
| 15 | 1.19 | 611.1502 | 611.1442 | 9.87 | $[M-H]^-$ | 306.0774、272.0908、128.0345、143.0462、288.0671、338.0506 | $C_{20}H_{32}N_6O_{12}S_2$ | Glutathione disulfide | Amino acids and their derivatives |
| 16 | 1.26 | 191.0202 | 191.0197 | 2.62 | $[M-H]^-$ | 111.0081、87.0085、85.0298、57.0330、67.0179 | $C_6H_8O_7$ | Citric acid | Organic acids |
| 17 | 1.30 | 111.0176 | 111.0186 | 9.01 | $[M-H]^-$ | 68.9961 | $C_4H_4N_2O_2$ | Uracil | Pyrimidines and pyrimidine derivatives |
| 18 | 1.34 | 119.0347 | 119.0340 | 5.46 | $[M+H]^+$ | 91.0436、77.0300、65.0312 | $C_4H_6O_4$ | Methylmalonate | Organic acids |
| 19 | 1.34 | 136.0595 | 136.0620 | 18.45 | $[M+NH_4]^+$ | 119.0206、94.0548、65.0325、77.0302 | $C_5H_6C_lN_5$ | Adenine hydrochloride | Others |
| 20 | 1.38 | 163.0391 | 163.0401 | 6.38 | $[M-H]^-$ | 119.0501 | $C_9H_8O_3$ | *p*-coumaric acid | Phenolic acids |
| 21 | 1.42 | 117.0193 | 117.0188 | 4.44 | $[M-H]^-$ | 73.0296、99.0089 | $C_4H_6O_4$ | Succinic acid | Organic acids |
| 22 | 1.42 | 282.0858 | 282.0848 | 3.44 | $[M-H]^-$ | 150.0419、133.0100、108.0205、107.0355 | $C_{10}H_{13}N_5O_5$ | Guanosine | Nucleosides, nucleotides, and analogues |
| 23 | 1.42 | 121.0509 | 121.0501 | 6.69 | $[M+H]^+$ | 77.0295、91.0433、93.0579 | $C_4H_8O_4$ | Erythrose | Saccharides |
| 24 | 1.69 | 221.0928 | 221.0930 | 0.90 | $[M+H]^+$ | 175.0878、157.0717、158.0582、130.0656 | $C_{11}H_{12}N_2O_3$ | 5-Hydroxy-L-Tr yptophan | Others |
| 25 | 1.84 | 283.0701 | 283.0679 | 7.77 | $[M-H]^-$ | 151.0250、108.0202 | $C_{10}H_{12}N_4O_6$ | Xanthosine | Nucleosides, nucleotides, and analogues |

| | | | | | | | | | |
|---|---|---|---|---|---|---|---|---|---|
| 26 | 2.07 | 137.0242 | 137.0244 | 1.31 | $[M-H]^-$ | 93.0352、65.0376 | $C_7H_6O_3$ | Salicylic acid | Phenolic acids |
| 27 | 2.26 | 147.0449 | 147.0452 | 2.04 | $[M-H]^-$ | 103.0544、77.0388 | $C_9H_8O_2$ | Cinnamic acid | Organic acids |
| 28 | 2.30 | 120.0810 | 120.0800 | 8.33 | $[M-H_2O+H]^+$ | 103.0543、93.0705、91.0546 | $C_8H_{11}NO$ | Phenylethanolamine | Others |
| 29 | 2.30 | 166.0866 | 166.0863 | 1.93 | $[M+H]^+$ | 120.0808、103.0544、93.0696、79.0552、121.0840 | $C_9H_{11}NO_2$ | L-Phenylalanine | Amino acids and their derivatives |
| 30 | 2.64 | 169.0151 | 169.0140 | 6.51 | $[M-H]^-$ | 125.023 | $C_7H_6O_5$ | Gallic acid | Phenolic acids |
| 31 | 2.64 | 331.0670 | 331.0649 | 6.31 | $[M-H]^-$ | 169.0156、125.0250、126.0290 | $C_{13}H_{16}O_{10}$ | Gallic acid hexoside | Tannins |
| 32 | 2.72 | 220.1178 | 220.1185 | 2.86 | $[M+H]^+$ | 90.0533、202.1101、184.0993、116.0305、124.0792、98.0235 | $C_9H_{17}NO_5$ | D-Pantothenic acid | Vitamin |
| 33 | 3.06 | 138.0545 | 138.0550 | 3.62 | $[M+H]^+$ | 120.0446、77.0389、65.0386、93.0560、92.0485、94.0664 | $C_7H_7NO_2$ | *p*-Aminobenzoic acid | Others |
| 34 | 4.10 | 206.1179 | 206.1176 | 1.65 | $[M+H]^+$ | 190.0838、162.0881、146.0589、145.0843、132.0786 | $C_{12}H_{15}NO_2$ | Dehydrosalsolidine | Alkaloids |
| 35 | 4.25 | 118.0647 | 118.0642 | 4.57 | $[M+H]^+$ | 91.0535、65.0375、117.0575 | $C_5H_{11}NO_2$ | Norvaline | Amino acids and their derivatives |
| 36 | 4.25 | 144.0796 | 144.0808 | 8.26 | $[M+H]^+$ | 143.0743、128.0472、127.0507、115.0536、117.0647、91.0546 | $C_{10}H_9N$ | 2-Naphthylamine | Others |
| 37 | 4.25 | 146.0597 | 146.0600 | 2.67 | $[M+H]^+$ | 91.0543、118.0648、117.0615、65.0383 | $C_9H_7NO$ | 3-Formylindole | Others |
| 38 | 4.25 | 205.0970 | 205.0972 | 0.63 | $[M+H]^+$ | 146.0597、188.0703、118.0636、132.0786、130.0637、144.0809、159.0909、170.0604 | $C_{11}H_{12}N_2O_2$ | L-Tryptophan | Amino acids and their derivatives |
| 39 | 4.29 | 134.0465 | 134.0477 | 9.25 | $[M-H]^-$ | 107.0365 | $C_5H_5N_5$ | Adenine | Others |
| 40 | 4.29 | 325.0960 | 325.0917 | 13.13 | $[M-H]^-$ | 119.0505、163.0401、164.0416 | $C_{15}H_{18}O_8$ | Coumaroyl hexoside | Phenolic acids |
| 41 | 4.29 | 474.1727 | 474.1729 | 0.44 | $[M+H]^+$ | 327.121 | $C_{20}H_{23}N_7O_7$ | Folinic acid | Others |

| | | | | | | | | | |
|---|---|---|---|---|---|---|---|---|---|
| 42 | 4.29 | 296.0844 | 296.0818 | 8.92 | $[M-H]^-$ | 134.0457、107.0346 | $C_{11}H_{15}N_5O_3S$ | 5'-Methylthioadenosine | Nucleosides, nucleotides, and analogues |
| 43 | 4.32 | 112.9856 | 112.9845 | 9.74 | $[M-H]^-$ | 68.9965 | $C_4H_2O_4$ | Acetylenedicarboxylic acid | Organic acids |
| 44 | 4.40 | 163.0402 | 163.0401 | 0.37 | $[M-H]^-$ | 119.0499 | $C_9H_8O_3$ | *m*-Coumaric acid | Phenolic acids |
| 45 | 4.63 | 299.1156 | 299.1136 | 6.62 | $[M-H]^-$ | 119.0490、59.0151、71.0143、83.0128、85.0280 | $C_{14}H_{20}O_7$ | Salidroside | Saccharides |
| 46 | 5.09 | 272.1223 | 272.1200 | 8.45 | $[M+H]^+$ | 161.0597、107.0483、123.0427、255.0968、237.0931 | $C_{16}H_{17}NO_3$ | Higenamine | Alkaloids |
| 47 | 5.78 | 193.0501 | 193.0505 | 2.38 | $[M-H]^-$ | 134.0374、178.0232 | $C_{10}H_{10}O_4$ | Ferulic acid | Phenolic acids |
| 48 | 5.85 | 293.1255 | 293.1237 | 6.04 | $[M-H]^-$ | 131.0704、101.0237、59.0133、143.0312、119.0328 | $C_{14}H_{18}N_2O_5$ | Glutamylphenylalanine | Amino acids and their derivatives |
| 49 | 6.12 | 314.1732 | 314.1750 | 5.82 | $[M+H]^+$ | 58.0653、1210640、175.0750、115.0518、122.0640 | $C_{19}H_{23}NO_3$ | N,O-dimethylcoclaurine | Alkaloids |
| 50 | 6.39 | 289.0735 | 289.0712 | 7.82 | $[M-H]^-$ | 245.0840、109.0298、123.0428、125.0226、187.0377、205.0498 | C15H14O6 | Catechin | Flavonoids |
| 51 | 6.49 | 289.0735 | 289.0712 | 7.82 | $[M-H]^-$ | 245.0840、109.0298、123.0428、125.0226、187.0377、205.0498 | $C_{15}H_{14}O_6$ | Epicatechin | Flavonoids |
| 52 | 7.00 | 207.0649 | 207.0652 | 1.45 | $[M+H]^+$ | 91.0532、147.0414、149.0211、119.0462、65.0382、53.0396 | $C_{11}H_{10}O_4$ | Citropten | Coumarins and derivatives |
| 53 | 7.04 | 223.0620 | 223.0607 | 5.96 | $[M-H]^-$ | 208.0393、193.0153、164.0476、149.0247、135.0443、121.0300、93.0350 | $C_{11}H_{12}O_5$ | Sinapic acid | Phenolic acids |
| 54 | 7.04 | 385.1171 | 385.1137 | 8.80 | $[M-H]^-$ | 223.0617、164.0480、149.0241 | $C_{17}H_{22}O_{10}$ | Sinapoylhexoside | Phenolic acids |
| 55 | 7.31 | 121.0288 | 121.0290 | 1.49 | $[M-H]^-$ | 92.0265、120.0230、93.0360 | $C_7H_6O_2$ | 4-Hydroxybenz | Others |

| | | | | | | | | | |
|---|---|---|---|---|---|---|---|---|---|
| | | | | | | | | aldehyde | |
| 56 | 7.62 | 286.1448 | 286.1440 | 2.69 | $[M+H]^+$ | 107.0489、143.0483、145.0614、209.0954、237.0822、238.0925 | $C_{17}H_{19}NO_3$ | N-Methylhigenamine | Alkaloids |
| 57 | 7.81 | 305.0715 | 305.0686 | 9.51 | $[M-H]^-$ | 96.9616、59.0150、225.1178 | $C_{12}H_{18}O_7S$ | Sulfo jasmonate | Lipids and lipid-like molecules |
| 58 | 8.38 | 300.1568 | 300.1600 | 10.66 | $[M+H]^+$ | 107.0490、269.1161、237.0914、175.0742、137.0587 | $C_{18}H_{21}NO_3$ | N-Methylisococlaurine | Alkaloids |
| 59 | 8.92 | 286.1432 | 286.1440 | 2.97 | $[M+H]^+$ | 107.0478、143.0479、137.0621、209.0960、269.1135 | $C_{17}H_{19}NO_3$ | Isococlaurine | Alkaloids |
| 60 | 9.41 | 300.1584 | 300.1600 | 5.26 | $[M+H]^+$ | 107.0486、143.0496、175.0733、237.0897、269.1126 | $C_{18}H_{21}NO_3$ | N-Methylcoclaurin | Alkaloids |
| 61 | 10.45 | 595.1645 | 595.1657 | 2.05 | $[M+H]^+$ | 325.0676、307.0617、361.0685、379.0726、391.0838、439.0777 | $C_{27}H_{30}O_{15}$ | Apigenin 6,8-digalactoside | Flavonoids |
| 62 | 10.56 | 163.0408 | 163.0401 | 4.23 | $[M-H]^-$ | 119.0492、90.0392、121.0286 | $C_9H_8O_3$ | *o*-Coumaric acid | Phenolic acids |
| 63 | 10.56 | 119.0502 | 119.0491 | 9.24 | $[M-H]^-$ | 91.0554、93.0346 | $C_8H_8O$ | 4-Vinylphenol | Others |
| 64 | 10.75 | 206.0822 | 206.0823 | 0.24 | $[M-H]^-$ | 164.0701、58.0303、91.0559、72.0087、70.0295 | $C_{11}H_{13}NO_3$ | N-Acetylphenylalanine | Amino acids and their derivatives |
| 65 | 11.02 | 611.3086 | 611.3100 | 2.31 | $[M+H]^+$ | 206.1177、489.2364、580.2678、568.2614、174.0908、107.0471 | $C_{37}H_{42}N_2O_6$ | Liensinine | Alkaloids |
| 66 | 11.06 | 563.1497 | 563.1406 | 16.16 | $[M-H]^-$ | 473.1154、443.1029、383.0712、353.0717、503.1252 | $C_{26}H_{28}O_{14}$ | Schaftoside | Flavonoids |
| 67 | 11.17 | 447.0980 | 447.1000 | 4.59 | $[M-H]^-$ | 327.0544、357.0644、429.0878 | $C_{21}H_{20}O_{11}$ | Isoorientin | Flavonoids |
| 68 | 11.52 | 300.1579 | 300.1600 | 6.90 | $[M+H]^+$ | 107.0486、143.0496、175.0733、237.0897、 | C1$_8H_{21}NO_3$ | N-Noramepavin | Alkaloids |

| | | | | | | | | | |
|---|---|---|---|---|---|---|---|---|---|
| | | | | | | 269.1126 | | e | |
| 69 | 11.59 | 286.1429 | 286.1440 | 3.84 | $[M+H]^+$ | 107.0483、269.1187、237.0905、238.1003、209.0985、143.0476 | $C_{17}H_{19}NO_3$ | Coclaurine | Alkaloids |
| 70 | 11.63 | 188.0353 | 188.0353 | 0.16 | $[M-H]^-$ | 144.0447、145.0495 | $C_{10}H_7NO_3$ | Kynurenic acid | Others |
| 71 | 11.63 | 165.0904 | 165.0910 | 3.57 | $[M+H]^+$ | 81.0702、109.1021、93.0686、67.0553、95.0451、91.0541 | $C_{10}H_{12}O_2$ | Phenethylacetat e | Others |
| 72 | 11.67 | 223.0974 | 223.0950 | 10.80 | $[M-H]^-$ | 179.1074、135.0865 | $C_{12}H_{16}O_4$ | Olivetolcarboxy lic acid | Phenolic acids |
| 73 | 11.67 | 609.1547 | 609.1461 | 14.02 | $[M-H]^-$ | 300.0284、301.0354、302.0430 | $C_{27}H_{30}O_{16}$ | Rutin | Flavonoids |
| 74 | 11.71 | 245.0938 | 245.0930 | 3.43 | $[M-H]^-$ | 20.0827、74.0243、116.0327、58.0289、70.0150 | $C_{13}H_{14}N_2O_3$ | N-Acetyltryptop han | Amino acids and their derivatives |
| 75 | 11.71 | 431.1031 | 431.1030 | 0.28 | $[M-H]^-$ | 311.0582、283.0628、341.0682、269.0459、293.0458 | $C_{21}H_{20}O_{10}$ | Vitexin | Flavonoids |
| 76 | 11.86 | 625.3261 | 625.3200 | 9.76 | $[M+H]^+$ | 206.1172 | $C_{38}H_{44}N_2O_6$ | Neferine | Alkaloids |
| 77 | 11.86 | 463.0941 | 463.0882 | 12.65 | $[M-H]^-$ | 300.0296、255.0321、271.0221、151.0019 | $C_{21}H_{20}O_{12}$ | Isoquercitrin | Flavonoids |
| 78 | 11.90 | 593.1594 | 593.1512 | 13.89 | $[M-H]^-$ | 285.0426、284.0330 | $C_{27}H_{30}O_{15}$ | Nicotiflorine | Flavonoids |
| 79 | 12.17 | 607.1377 | 607.1304 | 11.97 | $[M-H]^-$ | 300.0288、301.0351 | $C_{27}H_{28}O_{16}$ | Quercetin-3-O-p entosyl(1-2)acet ylpentoside | Flavonoids |
| 80 | 12.25 | 623.1722 | 623.1630 | 14.70 | $[M-H]^-$ | 315.0556、300.0311、299.0250、286.0504、287.0580 | $C_{28}H_{32}O_{16}$ | Isorhamnetin-3-O-rutinoside | Flavonoids |
| 81 | 12.40 | 447.0934 | 447.0985 | 11.34 | $[M-H]^-$ | 285.0354、285.0401、286.0424 | $C_{21}H_{20}O_{11}$ | Kaempferol-3-O -glucoside | Flavonoids |
| 82 | 12.40 | 461.0775 | 461.0725 | 10.78 | $[M-H]^-$ | 285.0412、286.0450、175.0231、113.0220 | $C_{21}H_{18}O_{12}$ | Kaempferol-3-O -glucuronoside | Flavonoids |
| 83 | 12.40 | 577.1618 | 577.1622 | 0.64 | $[M-H]^-$ | 269.0478、270.0480 | $C_{27}H_{30}O_{14}$ | Apigenin-7-neo | Flavonoids |

| | | | | | | | | | |
|---|---|---|---|---|---|---|---|---|---|
| | | | | | | | | hesperidoside | |
| 84 | 12.55 | 607.1762 | 607.1669 | 15.28 | $[M-H]^-$ | 299.0582、284.0346、285.0405、300.0605 | $C_{28}H_{32}O_{15}$ | Diosmin | Flavonoids |
| 85 | 12.59 | 453.1725 | 453.1700 | 5.54 | $[M+H]^+$ | 291.1291 | $C_{22}H_{28}O_{10}$ | 5-O-Methylvisammioside | Flavonoids |
| 86 | 13.05 | 431.1045 | 431.0984 | 14.29 | $[M-H]^-$ | 284.0345、285.0420、286.0470、256.0396、255.0337、227.0350 | $C_{21}H_{20}O_{10}$ | Afzelin | Flavonoids |
| 87 | 13.32 | 296.1628 | 296.1645 | 5.67 | $[M+H]^+$ | 235.0743、250.0940、265.1173、221.0936、207.0780、191.0895、179.0846 | $C_{19}H_{21}NO_2$ | Nuciferine | Alkaloids |
| 88 | 13.81 | 265.1421 | 265.1432 | 4.26 | $[M+H]^+$ | 187.1127、247.1308、229.1210、201.1253、163.0737、173.1315、135.0810、83.0494 | $C_{15}H_{20}O_4$ | Abscisic acid | Lipids and lipid-like molecules |
| 89 | 14.12 | 301.0357 | 301.0354 | 1.13 | $[M-H]^-$ | 178.9986、151.0038、121.0256 | $C_{15}H_{10}O_7$ | Quercetin | Flavonoids |
| 90 | 14.77 | 139.1110 | 139.1117 | 5.03 | $[M+H]^+$ | 69.0318、77.0370、83.0450、81.0685、91.0515、97.0631 | $C_9H_{14}O$ | Isophorone | Others |
| 91 | 14.92 | 327.2218 | 327.2178 | 12.13 | $[M-H]^-$ | 171.1041、229.1439、291.1910 | $C_{18}H_{32}O_5$ | (10E,15Z)-9,12,13-Trihydroxyoctadeca-10,15-dienoic acid | Lipids and lipid-like molecules |
| 92 | 15.46 | 329.2360 | 329.2334 | 7.78 | $[M-H]^-$ | 211.1345、229.1476、171.0098 | $C_{18}H_{34}O_5$ | (Z)-5,8,11-Trihydroxyoctadec-9-enoic acid | Lipids and lipid-like molecules |
| 93 | 15.57 | 130.0641 | 130.0646 | 3.77 | $[M-H_2O+H]^+$ | 103.0545、77.0389、102.0449 | $C_9H_9NO$ | Indole-3-carbinol | Others |
| 94 | 16.53 | 274.2730 | 274.2741 | 3.90 | $[M+H]^+$ | 57.0695、70.0649、88.0758、106.0870、274.2719 | $C_{16}H_{35}NO_2$ | Lauryldiethanolamine | Lipids and lipid-like molecules |

| | | | | | | | | | |
|---|---|---|---|---|---|---|---|---|---|
| 95 | 17.75 | 302.3044 | 302.3054 | 3.34 | $[M+H]^+$ | 284.2902、106.0822 | $C_{18}H_{39}NO_2$ | 2,2'-(Tetradecylimino)diethanol | Lipids and lipid-like molecules |
| 96 | 17.79 | 318.2991 | 318.3002 | 3.36 | $[M+H]^+$ | 60.0441、282.2765、300.2899 | $C_{18}H_{39}NO_3$ | Phytosphingosine | Lipids and lipid-like molecules |
| 97 | 18.17 | 675.3652 | 675.3589 | 9.30 | $[M-H]^-$ | 397.1330、277.2280、415.1514 | $C_{33}H_{56}O_{14}$ | DGMG 18:3 | Lipids and lipid-like molecules |
| 98 | 18.44 | 518.3219 | 518.3263 | 8.47 | $[M+H]^+$ | 184.0717、104.1077、86.0965 | $C_{26}H_{48}NO_7P$ | LPC 18:3 | Lipids and lipid-like molecules |
| 99 | 19.05 | 476.2855 | 476.2761 | 19.67 | $[M-H]^-$ | 279.2347、214.0500、196.0365 | $C_{23}H_{44}NO_7P$ | LPE 18:2 | Lipids and lipid-like molecules |
| 100 | 19.17 | 520.3384 | 520.3408 | 4.57 | $[M+H]^+$ | 184.0733、104.1052、86.0884、502.3319 | $C_{26}H_{50}NO_7P$ | LPC 18:2 | Lipids and lipid-like molecules |
| 101 | 19.32 | 293.2137 | 293.2122 | 5.18 | $[M-H]^-$ | 275.2017、231.2120、171.1012、 | $C_{18}H_{30}O_3$ | 9-HOTrE | Lipids and lipid-like molecules |
| 102 | 19.32 | 559.3189 | 559.3136 | 9.39 | $[M+HCOO]^-$ | 513.3126、514.3200、277.2188、253.0907 | $C_{27}H_{46}O_9$ | MGMG 18:3 | Lipids and lipid-like molecules |
| 103 | 19.36 | 331.2820 | 331.2840 | 5.92 | $[M+H]^+$ | 239.2352、95.0885、71.0855、257.2399、240.2388 | $C_{19}H_{38}O_4$ | Ceratodictyol | Lipids and lipid-like |

| | | | | | | | | | |
|---|---|---|---|---|---|---|---|---|---|
| | | | | | | | | | molecules |
| 104 | 19.55 | 452.2842 | 452.2773 | 15.19 | $[M-H]^-$ | 255.2336、256.2374 | $C_{21}H_{44}NO_7P$ | LPE 16:0 | Lipids and lipid-like molecules |
| 105 | 19.67 | 496.3388 | 496.3400 | 2.52 | $[M+H]^+$ | 184.0725、478.3257、104.1053 | $C_{24}H_{50}NO_7P$ | LPC 16:0 | Lipids and lipid-like molecules |
| 106 | 19.82 | 265.1502 | 265.1479 | 8.75 | $[M-H]^-$ | 96.9611 | $C_{12}H_{26}O_4S$ | Lauryl sulfate | Others |
| 107 | 19.93 | 478.2998 | 478.2942 | 11.67 | $[M-H]^-$ | 281.2505、196.0384、140.0111 | $C_{23}H_{46}NO_7P$ | LPE 18:1 | Lipids and lipid-like molecules |
| 108 | 19.97 | 295.2295 | 295.2278 | 5.49 | $[M-H]^-$ | 277.2188、195.1400、171.1040、113.0971 | $C_{18}H_{32}O_3$ | 9-HODE | Lipids and lipid-like molecules |
| 109 | 19.97 | 279.2302 | 279.2319 | 6.23 | $[M+H]^+$ | 95.0857、67.0540、81.0704、123.1151、173.1253、69.0693 | $C_{18}H_{30}O_2$ | Linolenic acid | Lipids and lipid-like molecules |
| 110 | 20.09 | 522.3527 | 522.3560 | 6.32 | $[M+H]^+$ | 184.0697、104.1104、505.3322 | $C_{26}H_{52}NO_7P$ | LPC18:1 | Lipids and lipid-like molecules |
| 111 | 20.16 | 561.3341 | 561.3290 | 9.14 | $[M+HCOO]^-$ | 279.2338、515.3256 | $C_{27}H_{48}O_9$ | MGMG 18:2 | Lipids and lipid-like molecules |
| 112 | 20.16 | 571.2958 | 571.2900 | 10.26 | $[M-H]^-$ | 255.2337、241.0132、314.8100 | $C_{25}H_{49}O_{12}P$ | LPI 16:0 | Lipids and lipid-like molecules |

| | | | | | | | | | |
|---|---|---|---|---|---|---|---|---|---|
| 113 | 20.85 | 597.3123 | 597.3046 | 12.97 | $[M-H]^-$ | 315.0527 | $C_{27}H_{51}O_{12}P$ | LPI 18:0 | Lipids and lipid-like molecules |
| 114 | 20.89 | 223.0627 | 223.0601 | 11.79 | $[M+H]^+$ | 207.0328、208.0341、209.0110 | $C_{11}H_{10}O_5$ | Isofraxidin | Coumarins and derivatives |
| 115 | 21.24 | 311.2037 | 311.2017 | 6.46 | $[M-H]^-$ | 149.0966 | $C_{21}H_{28}O_2$ | Norgestrel | Lipids and lipid-like molecules |
| 116 | 22.31 | 149.0442 | 149.0450 | 5.17 | $[M+H]^+$ | 133.0134 | $C_5H_8O_5$ | S-Citramalic acid | Lipids and lipid-like molecules |
| 117 | 22.31 | 355.2838 | 355.2843 | 1.46 | $[M+H]^+$ | 73.0462、81.0682、95.0846、107.0851、123.1169、175.1432、266.9954 | $C_{21}H_{38}O_4$ | Monolinolein | Lipids and lipid-like molecules |
| 118 | 22.38 | 326.3043 | 326.3054 | 3.28 | $[M+H]^+$ | 62.0602、326.2997、309.2715、308.2959 | $C_{20}H_{39}NO_2$ | Oleoyl ethanolamide | Lipids and lipid-like molecules |
| 119 | 22.92 | 714.5142 | 714.5043 | 13.84 | $[M-H]^-$ | 255.2333、279.2364 | $C_{39}H74NO_8P$ | PE 34:2 | Lipids and lipid-like molecules |
| 120 | 22.92 | 833.5243 | 833.5173 | 8.42 | $[M-H]^-$ | 241.0109、223.0007、255.2351、279.2353、391.2230、553.2806 | $C_{43}H_{79}O_{13}P$ | PI 34:2 | Lipids and lipid-like molecules |
| 121 | 22.92 | 282.2789 | 282.2800 | 3.90 | $[M+H]^+$ | 247.2401、265.2505、97.1012、111.1162、93.0855 | $C_{18}H_{35}NO$ | Oleamide | Lipids and lipid-like molecules |

| | | | | | | | | | |
|---|---|---|---|---|---|---|---|---|---|
| 122 | 22.96 | 738.5053 | 738.5031 | 2.91 | $[M-H]^-$ | 279.2352、458.2700、476.2760 | $C_{41}H_{74}NO_8P$ | PE 36:4 | Lipids and lipid-like molecules |
| 123 | 24.56 | 551.4198 | 551.4247 | 8.85 | $[M+H]^+$ | 175.1476、、135.1176、131.0841、119.0836、69.0727、81.0700 | $C_{40}H_{54}O$ | Echinenone | Lipids and lipid-like molecules |
| 124 | 25.22 | 568.4233 | 568.4300 | 11.70 | $[M]^+$ | 171.1180、147.1173、109.1020、105.0715 | $C_{40}H_{56}O_2$ | Lutein | Lipids and lipid-like molecules |
| 125 | 25.25 | 338.3410 | 338.3416 | 1.71 | $[M+H]^+$ | 321.3141、303.3026、57.0697、69.0694、83.0853、97.1010、81.0701 | $C_{22}H_{43}NO$ | Erucamide | Lipids and lipid-like molecules |

**Table S3.** The parameters of PLS-DA models.

| Ion mode | $R^2X$ | $R^2Y$ | $Q^2$ | $P_{CV\text{-}ANOVA}$ |
|---|---|---|---|---|
| Positive | 0.9167 | 0.9708 | 0.8768 | $8.58\times10^{-5}$ |
| Negative | 0.9200 | 0.9984 | 0.9184 | $1.18\times10^{-4}$ |

**Table S4.** The information of differential metabolites screened by PLS-DA.

| Metabolites | Formula | Compound class | VIP | P | Ion mode |
|---|---|---|---|---|---|
| Cinnamic acid | $C_9H_8O_2$ | Organic acids | 1.27 | $1.18\times10^{-3}$ | Positive |
| Fumaric acid | $C_4H_4O_4$ | Organic acids | 1.21 | $1.20\times10^{-4}$ | Negative |
| Rutin | $C_{27}H_{30}O_{16}$ | Flavonoids | 2.51 | $6.76\times10^{-6}$ | Positive |
| Schaftoside | $C_{26}H_{28}O_{14}$ | Flavonoids | 1.71 | $1.11\times10^{-2}$ | Positive |
| Afzelin | $C_{21}H_{20}O_{10}$ | Flavonoids | 1.25 | $2.82\times10^{-4}$ | Negative |
| kaempferol-3-o-glucuronide | $C_{21}H_{18}O_{12}$ | Flavonoids | 1.39 | $3.92\times10^{-5}$ | Negative |
| L-Phenylalanine | $C_9H_{11}NO_2$ | Amino acids | 5.23 | $1.11\times10^{-3}$ | Positive |
| L-Tyrosine | $C_9H_{11}NO_3$ | Amino acids | 1.03 | $3.24\times10^{-3}$ | Negative |
| Abscisic acid | $C_{15}H_{20}O_4$ | Lipids | 1.52 | $2.88\times10^{-5}$ | Positive |
| Oleamide | $C_{18}H_{35}NO$ | Lipids | 1.20 | $6.47\times10^{-5}$ | Positive |
| LPC16:0 | $C_{24}H_{50}NO_7P$ | Lipids | 1.43 | $5.33\times10^{-3}$ | Positive |
| LPC18:2 | $C_{26}H_{50}NO_7P$ | Lipids | 2.35 | $7.84\times10^{-4}$ | Positive |
| Indole-3-carbinol | $C_9H_9NO$ | Indole derivative | 1.58 | $1.43\times10^{-2}$ | Positive |
| Uridine | $C_9H_{12}N_2O_6$ | Pyrimidine | 1.91 | $3.36\times10^{-4}$ | Negative |
| *p*-Aminobenzoic acid | $C_7H_7NO_2$ | Benzoic acid derivative | 1.36 | $1.16\times10^{-2}$ | Positive |

**Table S5.** The linear equation, Regression coefficient, and Linear range of 13 standards.

| Compounds | Linear equation | Regression coefficient | Linear range (μg/mL) |
|---|---|---|---|
| Malic acid | y = 9101.7x - 759.66 | 0.9995 | 0.25-50 |
| Fumaric acid | y = 2197.9x - 1062.7 | 0.9956 | 1-20 |
| Gallic acid | y = 16342x - 46.854 | 0.9998 | 0.005-0.5 |
| Catechin | y = 2640.1x - 13.534 | 0.9997 | 0.005-0.5 |
| Epicatechin | y = 2353.5x - 7.8768 | 0.9998 | 0.005-0.5 |
| Schaftoside | y = 2698.7x + 23.166 | 0.9997 | 0.025-6 |
| Isoorientin | y = 11443x + 18.766 | 0.9997 | 0.005-0.5 |
| *p*-Coumaric acid | y = 21272x + 31.618 | 0.9999 | 0.005-1 |
| Sinapic acid | y = 1871.1x - 15.7 | 0.9999 | 0.025-8 |
| Ferulic acid | y = 709.49x - 5.1568 | 0.9992 | 0.025-4 |
| Rutin | y = 5056x - 61.122 | 0.9994 | 0.025-6 |
| Isoquercitrin | y = 4804.5x - 13.207 | 0.9993 | 0.005-1 |
| Cinnamic acid | y = 675.46x + 43.471 | 0.9986 | 0.05-20 |

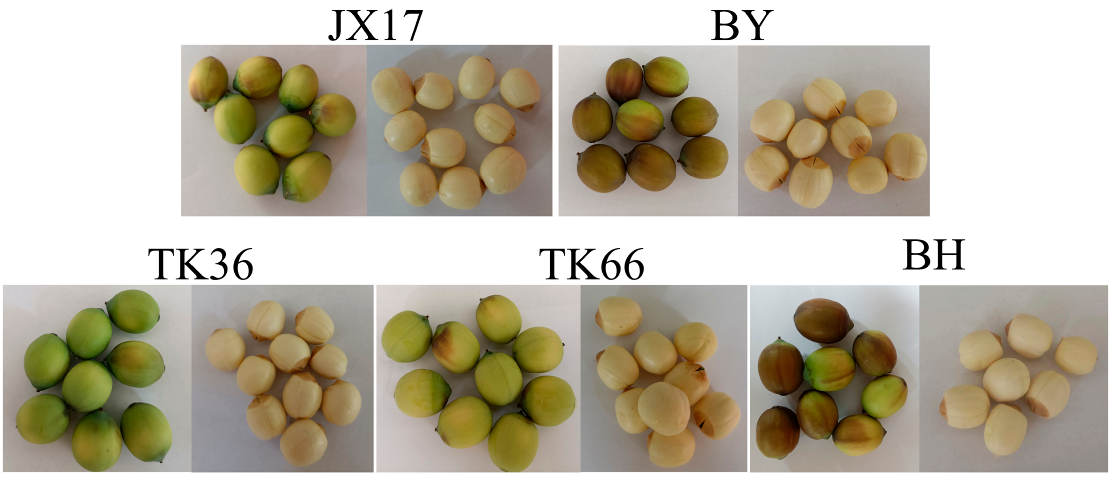


**Figure S1.** Appearance of different LS cultivars (left: with shell; right: without shell).

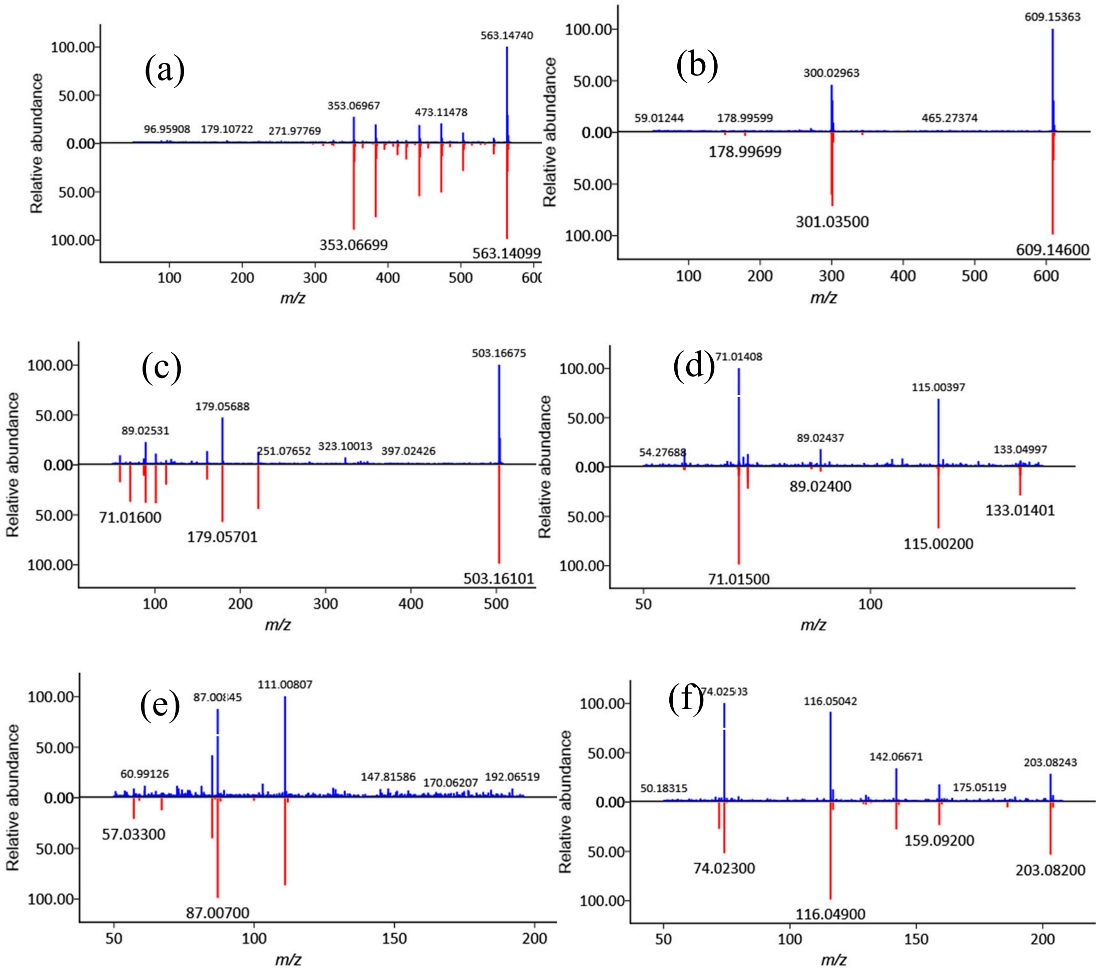


**Figure S2.** Selected-MS product ion spectra of Schaftoside (a), Rutin (b), Raffinose (c), Malic acid (d), Citric acid (e), and L-Tryptophan (f).

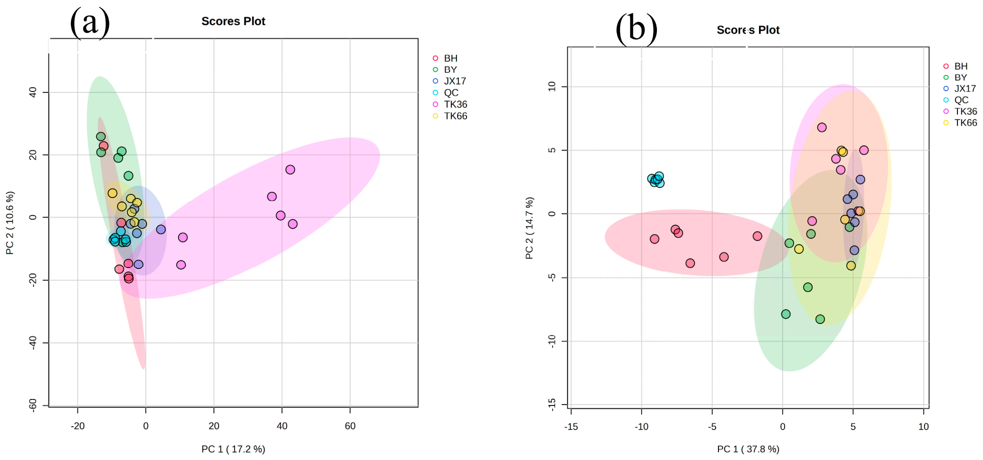


**Figure S3.** The PCA score plots for different LS cultivars in positive ion mode (a) and negative ion mode (b).
